# Receptive prosody adaptation to contextual feedback in autistic young adults

**DOI:** 10.3389/fpsyg.2026.1779814

**Published:** 2026-05-18

**Authors:** Chigusa Kurumada, Rachel Rivera, Christina Layton, Paul D. Allen, Loisa Bennetto

**Affiliations:** 1 Department of Brain and Cognitive Sciences, University of Rochester, Rochester, NY, United States; 2 Department of Psychology, University of Rochester, Rochester, NY, United States; 3 Department of Otolaryngology, University of Rochester Medical Center, Rochester, NY, United States

**Keywords:** adaptation, autistic young adults, discrimination, intonation, receptive prosody

## Abstract

**Introduction:**

Understanding spoken meaning requires adaptation to prosodic variability across speakers. Prior work shows autistic adolescents display reduced prosodic adaptivity despite intact perceptual sensitivity. The present study tested whether such reductions persist into adulthood.

**Methods:**

We recruited 69 autistic young adults (19–30 years) to complete an online pretest–training–posttest paradigm. Participants categorized utterances along a continuum from statement to question intonation, with feedback provided during training. A separate oddball discrimination task assessed baseline sensitivity. Mixed-effects logistic regression compared these data to previously collected groups (autistic adolescents, non-autistic adolescents, and non-autistic young adults).

**Results:**

Autistic young adults demonstrated overall adequate discrimination and significant adaptive shifts in categorization after training. However, the magnitude of adaptation was smaller than in non-autistic peers.

**Discussion/Conclusion:**

Together findings suggest that autistic adults can adapt to prosodic input but do so less, pointing to reduced perceptual learning based on contextual feedback rather than reduced perceptual acuity.

## 1 Introduction

Recognition of meaning is at the core of spoken language communication. Decades of research have shown that the social and perceptual profile of autism can affect effective meaning recognition (Kim et al., 2014; Siegal and Blades, 2003; Sturrock et al., 2022; Tager-Flusberg et al., 2005). Though the spectrum’s considerable heterogeneity resists broad generalization, even autistic children and adults without co-occurring language or cognitive disorders can often struggle to understand the meaning conveyed in speech (for reviews see Grice et al., 2023; Key and Slaboch, 2021; McCann and Peppe, 2003; Paul et al., 2005; Zhang et al., 2022). This difficulty is now widely recognized as a contributor to social isolation and academic or professional challenges.

A critical but often overlooked factor of this problem is the considerable variability in sound-meaning mapping in everyday language. Depending on factors such as their age, gender, and language background, speakers differ significantly in the way they produce units of meaning such as phonemes and words (e.g., Clopper and Pisoni, 2004; Goh, 2005; Kleinschmidt, 2019; Mullennix and Pisoni, 1990; Quam and Creel, 2021; Sommers and Barcroft, 2006). Effective comprehension must therefore be adaptive to accommodate these cross-speaker differences, and failure to do so can disrupt the communication of meaning (Bradlow and Bent, 2008; Kleinschmidt and Jaeger, 2015; Samuel and Kraljic, 2009, among others).

An influential experimental approach demonstrated this adaptivity by showing that listeners use contextual feedback to recalibrate their perception of ambiguous speech sounds (e.g., /t/ vs. /d/). In seminal work by Norris et al. (2003) and Kraljic and Samuel (2005, 2006), listeners were presented with a sound midway between two phonemes, embedded within words chosen so that only one interpretation was plausible (e.g., *cafe??eria*, which disambiguates the medial sound as /t/). Subsequently, listeners categorized more tokens from an acoustic /t/−/d/ continuum in the direction of the lexically supported phoneme, suggesting that they rapidly adapted their categorization function. Note that the adaptive shifts can encompass multiple sources of change, e.g., auditory-perceptual adjustments, distributional learning of cue distributions, and post-perceptual criterion shifts (for reviews, see Bent and Baese-Berk, 2021; Johnson and Sjerps, 2021; McMurray and Jongman, 2011; Xie et al., 2023). Notwithstanding the precise sources, the behavioral shifts have been robustly replicated across more than 300 experiments and have since become a standard tool for probing the rapid adaptation of behavioral responses, which allow listeners to navigate the variable speech input.

The variable mapping of sound to meaning may pose a unique challenge for autistic individuals, who typically seek precision and consistency in their experiences (Andreou et al., 2022; Baron-Cohen, 2009; Spackman et al., 2023). Their perceptual profiles are often described as highly veridical, meaning they are strongly influenced by the physical properties of sensory input and less adaptive to context or changes to it (Binur et al., 2022; Palmer et al., 2015; Turi et al., 2015, 2016). In various perceptual domains, such reduced adaptivity has been linked to atypical perceptual decision-making. For example, changes in lighting conditions in a room require adaptive changes in our internal computation of visual features (e.g., color, orientation, motion). However, autistic individuals tend to persist with fixed weights of sensory information, a factor now linked to hyper- and hyposensitivity to an environmental stimulus (Palmer et al., 2017; Sapey-Triomphe et al., 2021).

A recent study found that autistic adolescents exhibit reduced adaptivity in processing the prosodic variability of spoken language (Kurumada et al., 2024; for reviews on prosody in autism, see Eigsti et al., 2012; Gebauer et al., 2014; Grice et al., 2023; McCann and Peppe, 2003; Paul et al., 2005). Using a pretest–training–posttest design, Kurumada et al. (2024) tested three groups of participants: autistic adolescents, age-matched controls, and young adult controls. These participants were exposed to a prosodic continuum that gradually shifted from statements (e.g., “It’s raining”) to questions (e.g., “It’s raining?”). During the training phase, participants heard utterances that were midway between statements and questions and received feedback that disambiguated them as questions. While this training increased “question” responses to other prosodically ambiguous stimuli in the two control groups, the autistic adolescents showed significantly less adaptation. An additional perceptual discrimination task revealed equivalent levels of accuracy in all three groups, suggesting that the difference is specific to their adaptivity, not sensitivity, to the prosodic inputs (see also L. Wang et al., 2022).

If receptive prosody is less adaptive in autism, then this has significant theoretical implications. Previous research in this area has primarily focused on auditory perception (e.g., Can one hear subtle pitch contour differences?) and/or semantic-pragmatic comprehension (e.g., Can one comprehend the linguistic and affective meaning in context?) (for a recent, comprehensive review, see Grice et al., 2023). Rarely has it addressed the adaptive mapping between the two. This is a major gap in our knowledge because significant variability in everyday speech means that not all prosodic variations signify differences in meaning. Moreover, the relevant portion varies constantly from speaker to speaker. Consequently, enhanced pitch discrimination that is not calibrated well to a speaker can overwhelm—or even *hinder*—prosodic comprehension (Eigsti and Fein, 2013; Järvinen-Pasley et al., 2008). Alongside recent evidence of reduced flexibility and alignment in speech production (Hogstrom et al., 2022; Kissine et al., 2021; but also see Wehrle, 2023), reduced adaptivity in receptive prosody may provide a new link between communication difficulties in autism and their broader perceptual profiles.

The current study pursues a focused objective within this broader goal: identifying how listeners’ behavioral responses to prosodic inputs may change under feedback. We do so by following the standard of the field (e.g., Norris et al., 2003; Kraljic and Samuel, 2005, 2006)—short-term, criterion shifts as assessed by a possible shift of categorization function before and after the exposure. Also following the tradition of the field, we remain agnostic about the source of any detected differences. Specifically, our data cannot determine whether observed “adaptivity” reflects auditory/perceptual processing or it involves broader, task-dependent shifts in response strategy—a distinction that matters for precise understanding of how autism can affect speech perception and comprehension. The diagnosis remains a major challenge because successful adaptation likely draws on multiple interacting mechanisms (for a review and discussion, see Xie et al., 2023). Tracking a speaker’s voice alone requires integrating multiple levels of sensory input, sustained *and* flexible attention allocation (Alispahic et al., 2024), and audio-visual processing (e.g., tracking voices for different individuals); successful prosodic interpretation also requires communicative inference (e.g., “The speaker must have meant to ask a question”). Each of these processes and mechanisms can interact with autistic conditions, making any hasty causal explanation elusive.

Our aim here is therefore descriptive: to characterize behavioral signatures and determine how they differ, if at all, across (1) diagnostic groups (= autistic and non-autistic young adults) and (2) age groups (= autistic young adults and autistic adolescents). To do so, building upon the work of Kurumada et al. (2024), the current study provides new data from autistic young adults. This group was absent from the original study due to its strong focus on the younger population. Testing autistic adults in the same experimental context will help us determine two currently ambiguous possibilities. First, reduced adaptivity may be a chronic condition of autism that persists from adolescence into adulthood. In this case, the responses of autistic young adults should resemble those of autistic adolescents. Alternatively, reduced adaptivity could be a form of developmental delay, or the performance gap that may close over time with increased language exposure. In this case, the new results would resemble those of the control groups of non-autistic adolescents and young adults.

## 2 Methods

### 2.1 Participants

Sixty-nine autistic young adults aged 19–30 were recruited from SPARK research match (https://sparkforautism.org/) and participated in the experiment online in a self-paced manner. All participants were monolingual native speakers of American English with no known language disorder or intellectual disabilities. SPARK (Simons Foundation Powering Autism Research) is a large US autism cohort funded by the Simons Foundation, built in collaboration with over 31 university-affiliated research clinics across 26 states. SPARK eligibility requires a professional diagnosis of autism (e.g., by a clinical psychologist, pediatrician, or other specialty physician). All participants additionally self-reported a professional diagnosis of autism; however, the specific procedures and instruments were not reported. To account for the expected variability among participants given this heterogeneity as well as the relatively large age range (19–30 years), we increased the target group sample size by over 20% of the original study with adolescents (Table 1).^1^ We applied pre-determined screening measures with a threshold for autism characteristics on the Autism Spectrum Quotient (AQ) (Baron-Cohen et al., 2001) as well as task-internal attention check measures to ensure task engagement and stimulus audibility (for more details on participant characteristics, see Supplementary Information).

**Table 1:** Participant demographics.

Variable	Autistic		Non-autistic (control)	
	Adolescents	Young adults	Adolescents	Young adults
*N*	50	63*	50	50
Mean age in years	15.5 (1.33)	26.05 (3.15)	15.2 (1.45)	20.3 (2.59)
gender (female/male/nonbinary)	17/32/1	25/31/7	20/27/3	24/25/1
Professional diagnosis	100%	100%	0%	0%
SRS-2 total T-score	75.4 (1.2)	NA	43.9 (4.9)	NA
AQ total	NA	35.76 (6.53)	NA	NA

^*^Current data.

The number of participants in each group is for those included in the final analysis. Exclusions based on attention checks and discrimination accuracy were applied.

### 2.2 Procedure

The stimuli and procedure are the same as those used by Kurumada et al. (2024). See the Supplementary Information for details on the stimuli generation process. They were delivered through the same self-paced paradigm used for the control group of young adults. Participants received a link to the Gorilla.sc experimental platform (https://app.gorilla.sc/) and completed the tasks at home using their own computers and external listening devices, such as headphones or earbuds.^2^ The average total participation time for the experimental tasks was 17 min. To equate general participation experience across subjects, we planned to exclude participants whose time exceeded two SDs from the mean. However, no such cases occurred.

**Figure 1:**
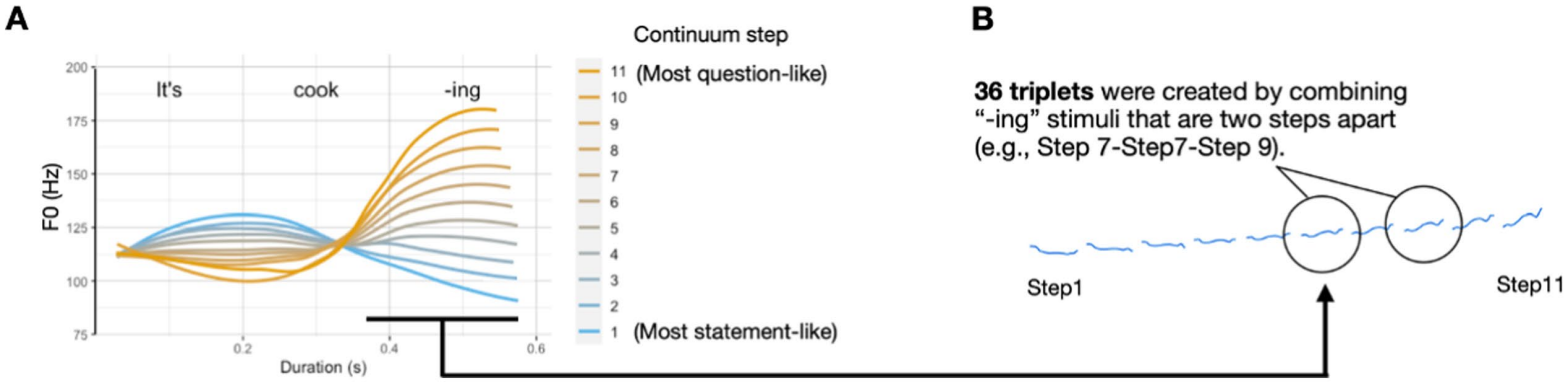
Stimuli for the adaptation and discrimination tasks. Diagrams regenerated based on Kurumada et al. (2024). **(A)** The 11-step continuum for “It’s cooking” used in the pre- and posttest blocks of the adaptation paradigm. The two endpoint stimuli were recorded by a male native speaker of American English in his twenties. The continuum was created by interpolating the mean fundamental frequency (*f*0) and duration of each syllable for nine intermediate steps. Details of the stimuli and their creation can be found in Xie et al. (2021). **(B)** Discrimination stimuli were created by concatenating the “ing” part of the continuum steps. Standard trials had three tokens of “ing” two steps apart (e.g., Steps 7–9), while suprathreshold trials had tokens six steps apart (Steps 3–9).

After a headphone check, participants first completed the adaptation task. This task employed an 11-step prosodic continuum ranging from declarative to interrogative intonation (e.g., “It’s cooking.” vs. “It’s cooking?”) generated through the manipulation of pitch and duration of natural utterances (Figure 1A). The stimuli were identical to those used in Kurumada et al. (2024), and the process of stimuli resynthesis is detailed in Xie et al. (2021) and repeated in the Supplementary Material Section 2. Participants completed three blocks: a pretest, a training session, and a posttest. The pretest and posttest each consisted of an identical set of 44 two-alternative forced-choice (2AFC) categorization trials (11 steps × 4 repetitions). Participants heard one token at a time and categorized its meaning as “asking” or “telling” by clicking a corresponding button on the screen. No feedback was provided in the pre- and posttests.

During the training block, participants responded to an additional 30 categorization trials, receiving feedback after each one. As in the test stimuli, the training stimuli took the form of “It’s X-ing,” where X was one of five different verbs: *booting, cooling, losing, muting, or moving*. Five continua were created in the same way as the test continuum and evaluated by 60 native American English speakers to determine the most ambiguous step in the asking-telling judgments. Fifteen tokens (50%; five verbs repeated three times each) were sampled from the lowest end of the continuum (i.e., the most statement-like) and accompanied by feedback stating that the speaker was “telling” something. The other 15 tokens were sampled from the midpoint, and the feedback stated that the speaker was “asking” something. This feedback appeared as printed text on the screen. A pleasant “ding!” tone accompanied by a smiley emoji appeared when the response corresponded to the intended mapping (“telling” after the lowest-end stimulus and “asking” after a midpoint stimulus). A low-pitched buzzer accompanied by a frowny-face emoji appeared when the response did not correspond to the intended mapping.

Note that the 30 training stimuli had five verbs with variable segmental features and included the long vowel (e.g., “cooling”) different from the short vowel (i.e., “cooking”) heard in the test. This was done to ensure that any observed adaptive shifts would not simply represent veridical acoustic memory traces of the training stimuli. Instead, for participants to show adaptive shift in their categorization judgments, they would need to extrapolate the *prosodic* features of the ambiguous stimuli and generalize this knowledge to the 11-step test contour, i.e., adapting to how the speaker produces question vs. statement contours.

Participants then completed a separate perceptual discrimination task, where triplets of spliced final syllables (“-ing”) from the continuum were presented in a 2AFC oddball format (Figure 1B).^3^ They were asked to identify either the first or the last item in a triplet as an oddball stimulus that differed acoustically from the others. A total of 72 tokens were created by pairing “-ing” tokens that were two steps apart (e.g., Step 1–3, 2–4, and 3–5, 36 triplets × 2 oddball options). An additional eight tokens included large pitch differences (six steps apart as opposed to two). These were used as suprathreshold tokens, which are very easy, to screen for basic perceptual acuity and task engagement. Participants who failed to achieve 75% accuracy on these tokens were excluded from final analysis. Four visual attention check trials were interleaved throughout the task to screen out anyone who did not achieve 100% accuracy on these trials, but no one was excluded from these trials. For details of the tasks, see Kurumada et al. (2024). All relevant stimuli, data, analysis and visualization scripts are downloadable from https://osf.io/p5v3k/.

### 2.3 Analysis overview and predictions

We analyzed data from the discrimination and adaptation tasks separately using mixed-effects logistic regression models (lme4 package) in the statistical programming language R (R Core Team, 2024). Specific fixed and random effects for each model are provided below.

In line with Kurumada et al. (2024), we applied a predetermined accuracy threshold based on suprathreshold (= very easy) discrimination trials to ensure that the adaptation results reflected differences in *adaptivity of categorization responses* rather than basic perceptual sensitivity or task engagement. In other words, the current discrimination task served as a screening measure to reduce baseline variability across compared groups, rather than to detect possible performance differences between groups and individuals. Based on this and prior work showing intact or heightened pitch sensitivity in autistic individuals, we expected all groups to have equivalent discrimination accuracy levels after the screening (Remington and Fairnie, 2017; Rotschafer, 2021).

For the main adaptation task, we examined the log odds ratio of “question” (vs. “statement”) responses during the pre- and posttests. By design, adaptation should result in an increase in “question” responses after training. Adaptive shifts are known to occur in both directions (i.e., question- vs. statement-biasing) in nonautistic young adults: After a brief exposure of about 4 min, utterances that are ambiguous between a question and a statement can be more easily interpreted as either (Xie et al., 2021). Kurumada et al. (2024) and the current study chose to target the *question-biasing* condition for two reasons. First, autistic children can sometimes show a baseline bias, responding “statement” to an utterance intended to be a question (Järvinen-Pasley et al., 2008). Although this bias may not persist into adulthood, it is deemed important to test whether contextual feedback can counteract it. Second, and relatedly, the utterances used in the current task have declarative syntax (e.g., “It’s cooking”). Learning to answer “statement” can be achieved by ignoring the intonation altogether. To test whether participants can recognize a speaker-specific pattern of intonation use, it is more informative to test whether they can learn to associate ambiguous utterances with the question meaning.

Our key prediction was that the block variable (pre- vs. posttest) would have a significant effect, which we tested in three steps. First, we analyzed the current data from autistic young adults to see if they demonstrated an adaptive shift in prosodic categorization. Second, we conducted a factorial analysis with those from the other three groups in Kurumada et al. (2024) to ask whether the degree of adaptivity varied across the two age groups (adolescents vs. young adults) and autism condition (autistic vs. non-autistic). Critically, if the reduced adaptivity found in Kurumada et al. (2024) persists into adulthood, we should observe a smaller pre- vs. post-block difference in responses in the autistic groups than in the control groups. Finally, we tested for individual differences in relation to participants’ age and, for the autistic adults, AQ scores. The study, however, was powered for group comparisons rather than individual-level inference. Moreover, the AQ was designed as a screening tool—not a diagnostic measure—and scores do not provide a linear index of autism severity (Ashwood et al., 2016; Lundqvist and Lindner, 2017). Accordingly, these final analyses are *post hoc* and exploratory.

## 3 Results

### 3.1 Screening based on the discrimination task

Six autistic young adult participants (8%) failed to reach the predetermined cutoff of 75% accuracy on the suprathreshold tokens and were excluded. This left 63 participants for further analysis.

**Figure 2:**
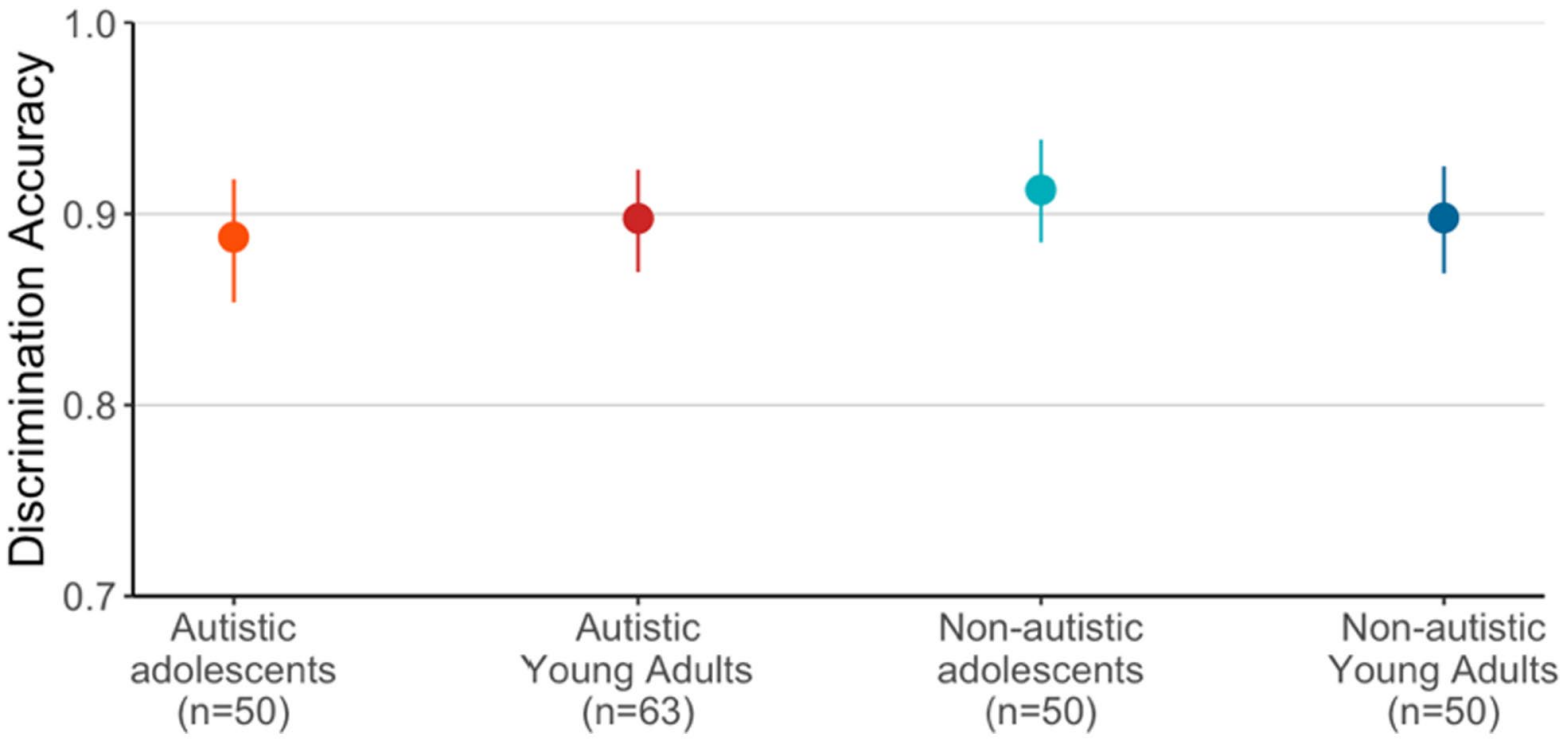
Mean accuracy of responses to the standard trials in the discrimination task by group. Error bars indicate bootstrapped 95% confidence intervals (CIs). Data shown here include only participants who exceeded the 75% accuracy threshold on the suprathreshold trials.

Overall accuracy was well above chance (50%) and consistently exceeded 85% for all groups, indicating that participants could reliably discriminate the acoustic differences in the stimuli (Figure 2). We used a generalized linear mixed model with a binomial family and a logit link function to analyze the data and examine the main effects of autism condition (factorial, contrast coded, non-autistic = −1 vs. autistic = 1), age group (factorial, contrast coded, adolescents = −1 vs. young adults = 1) and an interaction term between them. We used the maximal random effect structure justified by the data (i.e., by-participant intercepts and by-item intercepts) and slopes for the autism condition, age group, and their interaction (Barr et al., 2013). A model summary can be found in Supplementary Information.

As expected, neither of the main effects nor their interaction was significant (autism condition $\hat{\beta }$ = − 0.067, *z* = −0.604; age group $\hat{\beta }$ = − 0.131, *z* = −1.195; interaction $\hat{\beta }$ = 0.044, *z* = 0.408), suggesting that the four groups were equivalent in terms of their discrimination accuracy. These results support the feasibility of the adaptation task: Participants in the current study could accurately perceive and discriminate subtle shifts in prosodic contours from fall to rise. Importantly, the results underscore the assumption that any differences seen in the categorization and adaptation results are not solely attributable to perceptual differences.

### 3.2 Adaptation task results

**Figure 3:**
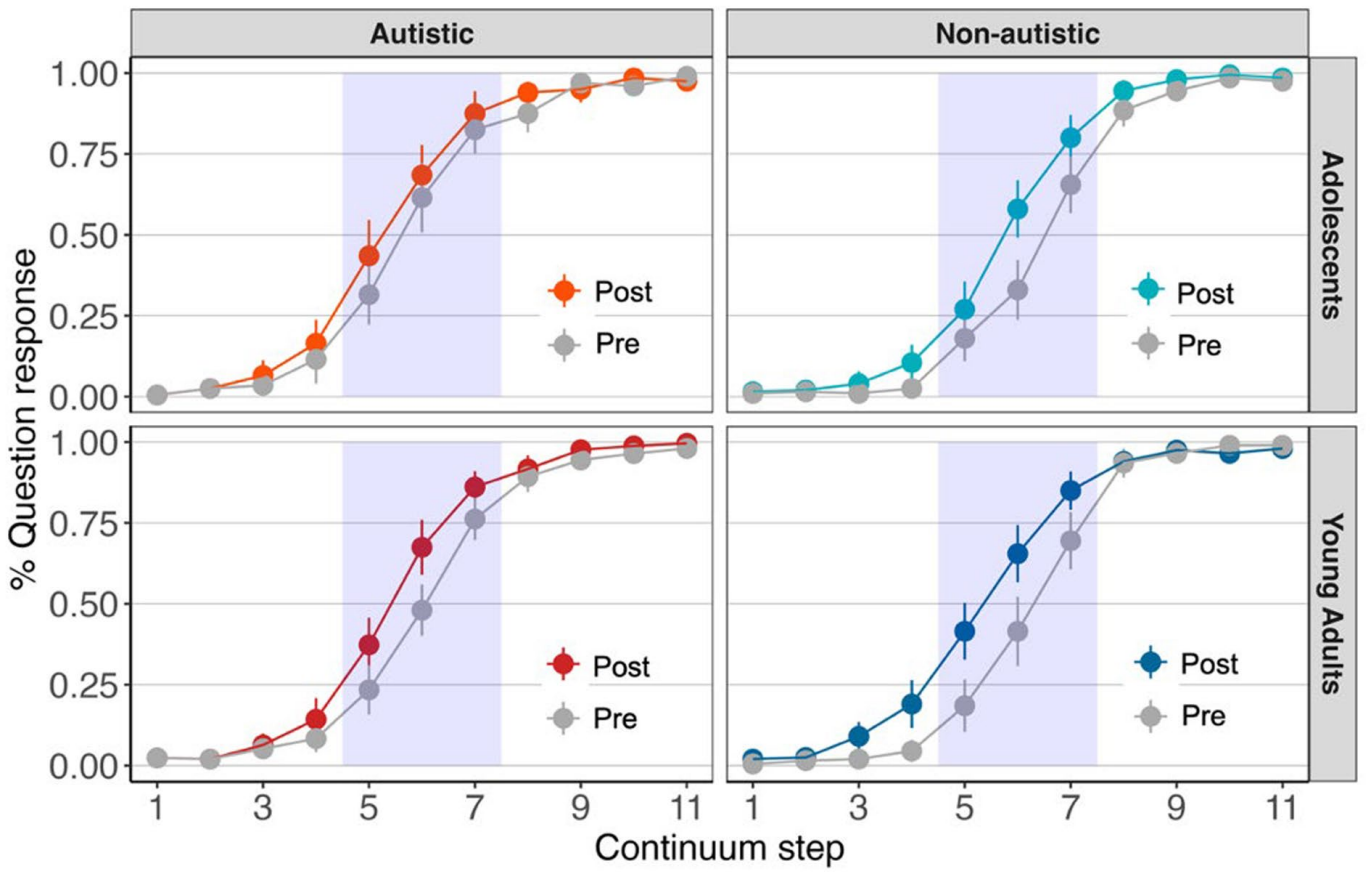
Proportions of responses to questions during pre- (gray) and posttest (colored) by group. Error bars indicate bootstrapped 95% confidence intervals (CIs). The light blue box in the background is added to each panel for visual inspection. It shows the mid-range (steps 5–7) of the continuum where the shift in response from pretest to posttest is expected to be most apparent. The factorial analysis on the effects of autism and age group targeted the data points from here.

Figure 3 shows the proportion of “question” responses in the pre- and posttest blocks. The responses from the autistic young adults replicated the previous results with autistic adolescents in two ways. First, steps 1 and 11 were reliably identified as statements and questions, respectively. This means that, when the contours clearly fell or rose, the prosody-meaning mapping was easily recognizable. Second, the categorization function from the pre- vs. posttest showed the predicted leftward (upward) shift after training, suggesting that the training led to adaptive shifts of responses. However, the magnitude of the shift appears to be smaller compared to the two control (non-autistic) groups. As in the discrimination task, we used a generalized linear mixed-effects model with a binomial family and a logit link function. We examined the data in three steps as described in Section 2.3.

Autistic young adult data: Do autistic young adults show receptive prosody adaptation? Using the current data, we considered two fixed effects and their interaction term: block (factorial; contrast coded: posttest = 1, pretest = −1) and continuum (numerical variable: 1–11; centered). Centering the continuum ensures that its interaction with the other two fixed effects is assessed at the center of the continuum, where the adaptation effect is expected to be most prominent. We used the maximal random effect structure justified by the data, i.e., by-participant random intercepts and slopes for block and continuum and the interaction between them.

Table 2 provides the model summary. Besides the expected effects of continuum ( $\hat{\beta }$ = 1.497, *z* = 17.043, *p* < 2e−16), we found a strong main effect of block ( $\hat{\beta }$ = 0.503, *z* = 5.189, *p* < 0.0001). That is, autistic young adults showed a significant increase in the likelihood of question responses *post* training, i.e., adaptation. The significant continuum × block interaction means that items with a clear rising contour were even more reliably categorized as questions in posttest than would be expected from the overall adaptive shift of responses ( $\hat{\beta }$ = 0.013, *z* = 2.363, *p* < 0.02).

**Table 2:** Fixed-effect estimates of the generalized linear mixed-effect model predicting the question response in the autistic young adult data by block and continuum.

Predictor	Estimate	Std. error	*z* value	Pr (>|*z*|)	
(Intercept)	0.54	0.191	2.822	0.004	^**^
Block (posttest = 1)	0.503	0.097	5.189	2.11e−07	^***^
Continuum steps	1.497	0.087	17.043	<2e−16	^***^
Block * Continuum	0.013	0.054	2.363	0.018	^*^

*** *p* < 0.001; ** *p* < 0.01; * *p* < 0.05.

**Figure 4:**
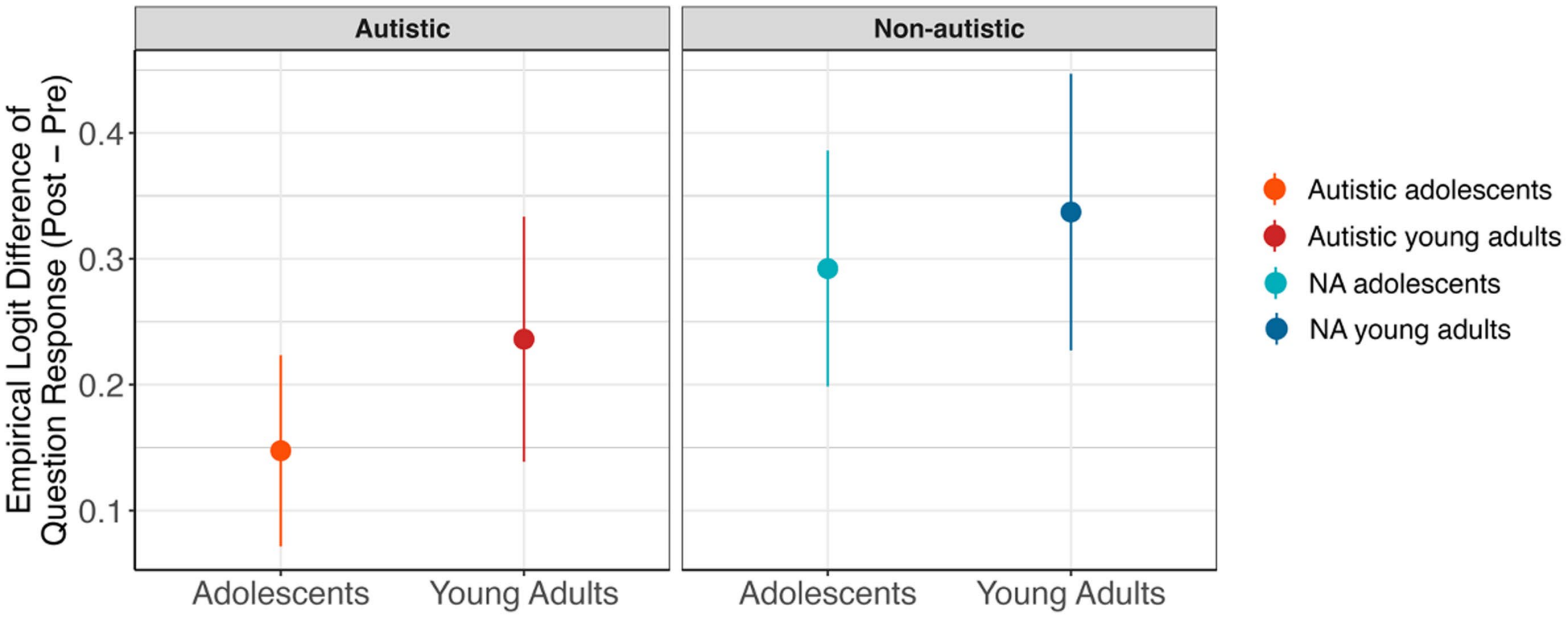
Empirical logit transformed difference of question responses computed by subtracting the average in pretest from the average in posttest. This represents the level of adaptivity, as operationalized as the degree to which question responses increased (or decreased) after training compared to before. Error bars indicate bootstrapped 95% confidence intervals (CIs).

Autism × age: Does adaptivity change according to autism condition and age? We focused this comprehensive analysis on the continuum midpoint (Steps 5–7), where adaptive shifts are most detectable, avoiding a hard-to-interpret four-way interaction over all steps. For the sake of completeness, we conducted a full-continuum analysis (see Supplementary Materials) and confirmed that the results were consistent with what we present below. Figure 4 shows pre–post differences in the likelihood of question responses across the four groups, expressed as empirical logits to handle bounded proportions and unequal variance. A value of zero indicates no adaptation. Visual inspection suggests greater adaptation in non-autistic than autistic participants, and slightly greater adaptation in young adults than adolescents within each group.

Our new mixed-effects regression model included three main effects and their interactions: autism condition (factorial; contrast coded: non-autistic = −1, autistic = 1), age group (factorial; contrast coded: adolescents = −1, young adults = 1), and block (factorial; contrast coded: pretest = −1, posttest = 1). As above, we used the maximal random effect structure justified by the data, i.e., by-participant random intercepts and slopes for block.

Table 3 provides the model summary. The expected effect of block ( $\hat{\beta }$ = 0.375, *z* = 9.69, *p* < 2e−16) was significant. This means that participants were overall more likely to respond “question” post training, i.e., adaptation. This is after accounting for the autistic participants’ bias towards responding question ( $\hat{\beta }$ = 0.246, *z* = 3.122, *p* < 0.002), which is even stronger in the autistic adolescent population (i.e., autism × age interaction: $\hat{\beta }$ = − 0.174, *z* = −2.211, *p* < 0.03).

**Table 3:** Fixed-effect estimates of the generalized linear mixed-effect model predicting the question response by autism condition, age group, and block.

Predictor	Estimate	Std. error	*z* value	Pr (>|*z*|)	
(Intercept)	0.267	0.079	3.382	0.0007	^***^
Autism (autistic = 1)	0.246	0.078	3.122	0.001	^***^
Age group (young adults = 1)	0.0004	0.078	0.005	0.995	
Block (posttest = 1)	0.375	0.038	9.690	<2e−16	^***^
Autism × Age group	−0.174	0.078	−2.211	0.027	^*^
Autism × Block	−0.079	0.038	−2.069	0.038	^*^
Age group × Block	0.063	0.038	1.654	0.098	
Autism × Age group × Block	−0.003	0.038	−0.083	0.933	

The analysis focuses on the data from the middle steps (5, 6, and 7). Significance codes: *** *p* < 0.001; ** *p* < 0.01; * *p* < 0.05.

With respect to adaptation, two key effects emerged. First, and critically, autistic participants adapted less than non-autistic participants (Autism × Block: $\hat{\beta }$ = − 0.079, *z* = −2.069, *p* < 0.04; Figure 4). That is, in each age group (adolescents, young adults), autistic participants showed a smaller increase of “question” responses post training. Second, a marginal age group × block interaction ( $\hat{\beta }$ = 0.063, *z* = 1.654, *p* < 0.099) suggests slightly greater adaptation in young adults than adolescents within each diagnostic group, though wide within-group variance (bootstrapped 95% CIs in Figure 4) warrants caution. The three-way interaction was not significant ( $\hat{\beta }$ = − 0.003, *z* = −0.083), indicating a similar developmental trend across autistic and non-autistic groups.

In summary, the planned main analyses of autism diagnosis and age yielded two key findings. When analyzed independently, autistic young adults showed a significant adaptive shift in their categorization responses. This provides new evidence that their receptive prosody is sensitive to contextual feedback and adapts to it after four to 6 min of exposure to a new speaker and feedback on their intended prosody-meaning mapping. However, when compared to corresponding NA groups, the degree of adaptivity was smaller. A marginal interaction of age and block suggests possible maturational changes within both the autistic and NA participants.

Individual differences (exploratory analyses): To better understand the links between adaptivity and autism, we ultimately seek how autism symptomatology and maturational factors predict greater or lesser adaptivity. Here we provide two *post hoc*, preliminary examinations. The first examined participants’ numerical age rather than categorizing them as adolescents or young adults. The second targeted only autistic young adults and investigated whether their AQ scores predicted adaptation. (This information was available only for the autistic young adults.) Details of the regression models and summary tables of fixed effects are provided in Supplementary Information.

To assess the gradient effect of age, we updated the above holistic model and replaced the categorical age group variable with a numeric age variable. The rest of the model specifications remained the same. We found no main effect of age ( $\hat{\beta }$ = 0.002, *z* = 0.121). Neither the two-way interaction with block ( $\hat{\beta }$ = 0.01, *z* = 1.117) nor the three-way interaction with autism and block was significant ( $\hat{\beta }$ = −0.009, *z* = −0.962). This could be attributable to the relatively small age ranges of the adolescent group (ages 13–17, mean = 15.5, standard deviation = 1.42) and the non-autistic young adult group (ages 18–24, mean = 20.3, standard deviation = 2.59), which limits statistical power. Future studies must expand the age ranges to assess additional maturational changes.

Finally, we modeled the effects of AQ total scores. This model used data from autistic young adults only and included two fixed effects and their interactions: block (factorial; contrast coded: posttest = 1, pretest = −1) and AQ total score (numeric, centered). We used the maximal random effect structure justified by the data, i.e., by-participant random intercepts and slopes for block. Neither the AQ scores ( $\hat{\beta }$ = − 0.028, *z* = −1.432) nor their interaction with block ( $\hat{\beta }$ = −0.007, *z* = −0.663) were significant. In sum, the group-level finding of reduced adaptivity was not modulated by AQ score within the autistic group, suggesting that the adaptation difference between groups is not simply a function of autism trait severity as measured by this instrument. This, however, merits further investigation with finer-grained diagnostic measures of autism.

## 4 General discussion

Understanding everyday speech requires both accuracy and adaptivity in perception: The same prosodic contour can signal different meanings depending on speaker and context, while acoustically and prosodically distinct contours may convey similar meanings. This study provides novel evidence that autistic young adults adapt their receptive prosody categorization to contextual feedback, though to a lesser degree than non-autistic controls. Importantly, as in Kurumada et al. (2024), we focused our analysis on autistic young adults who passed the perceptual screen and demonstrated discrimination accuracy. This helps to bolster the conclusion that the group difference lies in adaptivity rather than in the ability to perceive an intonation contour *per se*.

Our findings indicate that autistic adults *do* adapt their receptive prosody categorization in response to contextual feedback—consistent with recent work showing adaptive learning at other levels of speech. For instance, Cummings et al. (2025) addressed this question at the phoneme level, using a fricative contrast (“sign” vs. “shine”). They demonstrated that repeated exposure to a sound ambiguous between “s” and “sh” in a lexical context was sufficient to trigger adaptive perceptual shifts (e.g., “dino/?/aur,” categorized as “s”). Similarly, Bestelmeyer et al. (2018) successfully elicited aftereffects to vocal expressions of emotions (angry vs. fearful) and phonemes (“m” vs. “o”). Aftereffects refer to changes in neural firing, and therefore sensitivity, due to continued stimulation or exposure, and this phenomenon has been argued to be diminished in autistic children aged 8–13 (Pellicano et al., 2007), lending further context to the pattern we observe.

We believe that successful *prosodic* adaptation is particularly noteworthy because prosodic contrasts are less categorical than phonetic ones and rely on broader contextual interpretation (e.g., distinguishing a question from a statement). To calibrate their receptive prosody to feedback, a listener must rely on a global understanding of conversational contents, and what an interlocutor intended to communicate via a chosen set of words and a holistic intonation contour. That autistic young adults adapt at this level indicates a capacity to manage the gradient and context-dependent nature of prosodic categories. Of note, however, is that the *magnitude* of adaptation was consistently smaller in autistic individuals than in their non-autistic peers in the current results. Thus, the adaptivity critical in receptive prosody appears to be reduced, if present.

Our multi-age design also illuminates possible developmental changes in adaptivity as we studied here. Two classes of possibilities are of promise and importance. One pathway implicates cognitive control. Adaptation requires tracking dynamic shifts in prosodic features across syllables, mapping them onto memory representations, and adjusting categorization based on feedback (e.g., Bent and Baese-Berk, 2021; Samuel and Kraljic, 2009; Sohoglu and Davis, 2016; Xie et al., 2023) Naturally, adaptivity is known to vary with individuals’ linguistic and cognitive abilities, in particular with the ability to navigate working memory load (Davidson and Souza, 2024; McLaughlin et al., 2018). These perceptual and attentional demands may overload adolescents but may be more manageable for younger adults (X. Wang et al., 2017). Adult listeners may also be better equipped to devise compensatory strategies that improve their task navigation (Eigsti et al., 2016).

The second pathway involves exposure to increased variability in speech input. At its core, adaptation requires updating prior expectations to align with current input, making initially surprising or ambiguous acoustic-phonetic input more predictable and meaningful. Listeners with prior experience that matches the current input have been shown to adapt more efficiently (e.g., Bradlow and Bent, 2008; Hanulíková and Levy, 2025; Porretta et al., 2016; Witteman et al., 2013). Conversely, limited social exposure, which is common among autistic children and adults, can result in sparse and potentially less varied prior prosodic experiences (an idea originally presented by Alispahic et al., 2022). Adaptivity in receptive prosody may thus be dampened by the restricted nature of the input but could potentially be strengthened by expanding the social network and increasing exposure variability (Kutlu et al., 2024).

Together, these considerations call for a multifactorial account of prosodic adaptation in autism, which is likely shaped both by the input and constraints of an immediate task environment as well as by long-term linguistic and social experiences. Research is increasingly examining early prosodic input in the home and its potential links to autistic children’s language development (Fusaroli et al., 2023; Pierce et al., 2023; Swanson et al., 2018; Woolard et al., 2023). Similar large-scale, dense sampling of linguistic input will illuminate the types and amounts of cross-talker prosodic variability in the input to autistic children and adolescents, which could inform the degree of adaptivity required for perception (Kleinschmidt, 2019). In addition to interpersonal communication, passive or non-interactive media exposure increases during adolescence (Beccaria et al., 2024), and its impact on adaptation requires further investigation. Combining such ecological data with experimental tests will help to clarify how atypical patterns of perceptual decision-making can emerge from the complex interplay of neurobiological and experiential factors characteristic of autism (Lawson et al., 2014; Mottron et al., 2006; Palmer et al., 2017; Soulières et al., 2007).

We acknowledge that the current sample is highly selective—verbally fluent, perceptually acute, and task-engaged—and therefore skewed toward the higher end of the linguistic and cognitive ability spectrum. This was a deliberate choice to ensure reliable execution and interpretation at this initial stage of investigation, but it inevitably limits generalizability. A further constraint is that the task presented a binary choice between a question and a statement, requiring relatively categorical response shifts rather than the more gradual adjustments characteristic of naturalistic prosodic interpretation in an ecological context. As such, the reduced criterion shift observed in the autistic group may reflect not strictly perceptual adaptivity but also cognitive control demands — such as attention switching, response perseveration, greater reliance on prior expectations, or reduced tolerance for conflicting information (Demetriou et al., 2018; Filipe et al., 2018; Palmer et al., 2017).

Expanding future samples to include more linguistically and cognitively diverse populations, alongside control tasks targeting specific cognitive factors, will be essential to characterizing the full range of profiles that shape prosodic adaptivity. Note that in our current autistic adult sample, there was no clear trend between AQ scores and adaptation likelihood. This may be due in part to the fact that, while the AQ is useful as a screening tool (Ashwood et al., 2016), it may be limited in its ability to explain variability within groups of diagnosed individuals. Alternatively, the perceptual and cognitive characteristics of autism may affect adaptation in multiple ways, defying any simple correlation between the two metrics. Previous studies have suggested that speaker-specific adaptation can occur at earlier auditory processing stages (Huang and Holt, 2009; Laing et al., 2012; Liu and Holt, 2015) and at the level of more abstract phonology-meaning contingencies (e.g., How likely will the current speaker produce a particular prosodic contour to signal a contrastive meaning? Roettger and Rimland, 2020).

Additionally, cross-modal comparisons, such as those between prosodic adaptation and adaptation in visual motion perception or multisensory integration, could clarify whether reduced adaptivity is general to all domains or specific to speech. This knowledge is key to effective diagnosis and intervention. If the reduction in adaptivity tested in the current paradigm is pervasive across domains of information processing, then the current results may predict a range of perceptual atypicality (e.g., adaptivity in visual motion processing). Conversely, if reduced adaptivity is domain-specific, relevant interventions should target auditory and linguistic processing, as well as the variability of speech sounds present in daily communication environments.

Finally, collaboration with autistic self-advocates will provide essential insight into lived communication experiences (e.g., Cummins et al., 2020; Nicolaidis et al., 2015). The experiment presented here used isolated utterances with clear feedback to provide a circumscribed example of adaptation that supports communication. However, autistic young adults’ responses to our post-experimental questionnaire revealed that even this simplified example vividly evoked many real-life instances of tuning into different speech patterns and styles. Misaligned expectations and communicative breakdown seem to be deeply felt in everyday interactions, underscoring the importance of further research on this topic and deeper understanding of when and how prosodic variability creates barriers to autistic individuals. To this end, we conclude with two quotes from our post-experiment questionnaire in which participants described their difficulty understanding someone’s message based on their tone of voice.

I experience, sometimes an annoying amount of, clarifying what I or others meant conversations throughout my daily life as a result of not understanding others’ tones/nonverbal cues, as well as a result of mine not matching what others expect.… I have had people mad at me because I didn’t reply because I didn’t understand that they were asking me something. I’ve also had people annoyed that I’m redundantly answering their statement.

This mostly comes up at work where I struggle sometimes to understand if someone is asking me a question or needs my help and either I provide help when it is not needed (which is offensive) or I ignore plea for help when it is needed (also offensive). I struggle with understanding whether someone needs something from me or not. Am I answering a question you are asking or am I just listening to you?

## Data Availability

The original contributions presented in the study are included in the article and Supplementary material. All stimuli, data, analysis, and visualization scripts are also publicly available at https://osf.io/p5v3k/.

## Footnotes

1 The two young adult groups have slightly different age ranges, with the autistic young adults spanning a wider range. This reflects recruitment challenges and the desire to maximize sample size, while the control group was drawn from the original study with slightly different recruitment parameters (e.g., controlling for their educational and geolocational profiles). We note that previous studies using the current task and stimuli with a comparable age range of NA adults produced data patterns indistinguishable from those of the current NA young adult group (Kurumada and Buxó-Lugo, 2024; Ostrow and Kurumada, 2026; Xie et al., 2021). We therefore do not analyze the range difference as a predictor. It is, however, possible maturational and age-related changes during adolescence–adulthood are of interest in adaptive speech perception and beyond (Mousley et al., 2025). We thank an anonymous reviewer for directing our attention to this point.

2 At the outset of the experiment, participants were asked to (1) turn off all notifications on their phone and computer, (2) move to a quiet room with minimal background noise, and (3) wear an external listening device. They were prevented from moving to the next page until explicitly verifying all three of these conditions. Prior to the experimental trials, participants adjusted their listening level using a calibration sound file. The file consisted of a stereophonic musical tone (a ringtone excerpt), encoded as a stereo MP3 at a sampling rate of 48,000 Hz. The file had an integrated loudness of −23.7 LUFS (Loudness Units relative to Full Scale), measured according to the EBU R128 standard, a loudness range (LRA) of 8.8 LU, and a true peak level of −9.0 dBFS. This matches the rest of the sound stimuli used in the experiment. Participants were instructed to adjust their playback volume until they perceived this sound at a comfortable listening level, and this volume setting was then maintained throughout the experiment without further adjustment.

3 The specific stimulus design in Kurumada et al. (2024) was based on three main factors. First, speech-based stimuli were selected over other options (e.g., musical or pure tones) due to well-documented speech-specific deficits in autism. To test participants’ ability to discriminate subtle differences in intonation, acoustically and indexically rich human speech stimuli were used. Second, the brevity of the “-ing” stimuli reduced the task difficulty for all participants. Memorizing and judging three instances of a sentence-long intonation contour (e.g., for “It’s cooking”) would tax working memory capacity, which could unfairly load onto stimulus discrimination in autistic participants. Third, the task aimed to elucidate basic perceptual sensitivity rather than the linguistic categorization of speech sounds. Using a content word, such as “cooking,” would blur the boundary between the two because it likely evokes lexical and exemplar-driven processes. The semantic opacity of “-ing” was expected to minimize this possibility. For the sake of direct comparison, we reused the original stimuli and procedure.

## Supplementary material

The Supplementary material for this article can befound online at: https://www.frontiersin.org/articles/10.3389/fpsyg.2026.1779814/full#supplementary-material
