# Supplementary material for: Receptive prosody adaptation to contextual feedback in autistic young adults
Source: Front Psychol. 2026 May 18;17:1779814. doi: 10.3389/fpsyg.2026.1779814 (PMC13261718; doi:10.3389/fpsyg.2026.1779814)
Supplement: Supplementary file 1 [file Table_1.DOCX]

*Supplementary Material for “Receptive Prosody Adaptation to contextual feedback in Autistic Young Adults”*

# Participant screening and steps taken to control the data validity

## Our participants were recruited from the SPARK Research Match database (https://www.sfari.org/resource/research-match/). The initial invitation email was sent to eligible young adults who (a) have a professional diagnosis of autism; (b) were aged 18–35; and (c) grew up hearing and speaking only American English. We confirmed their monolingual status using an additional screening question. Participants were also notified that they must be able to participate in the study independently in a quiet place with minimal background noise and that they must use external listening devices, such as earphones or a headset.

## Participant Screening Based on Autism Conditions

## We administered the Autism Spectrum Quotient (AQ) questionnaire as an additional screening measure (Baron-Cohen et al., 2001). We admitted participants with a total score above the threshold of 26. We also obtained information about the participant's age of and type of professional who gave them their diagnosis.

## Participant Exclusion Due to Task-related Behavioral and Attentional Difficulties

We used a set of predetermined criteria to exclude participants whose behavioral and attentional difficulties would significantly interfere with the administration process. This was done before data analysis. A similar set of criteria was used in the original study, and Kurumada et al. (2024) report on the number of participants removed according to each criterion in the supplementary information. Among the autistic young adults who participated in present study, **none were excluded at this step**.

1. Inability to achieve a score of at least 75% on the visual attention check trials. During these trials, participants saw three geometric shapes (e.g., a circle, a triangle and a diamond) in three different colors. Written instructions specified a target (e.g., “Click on the red circle to continue”). Four such trials were scattered across the discrimination task.
2. Clear evidence of not responding to the acoustic properties of the stimuli (e.g., invariant responses throughout each task, or systematic left- or right-sided bias in responding).
3. Significant time gaps (more than 10 minutes between tasks, or more than three minutes within a task), or total participation time for the two tasks exceeding two standard deviations from the mean (17 minutes).
4. Self-reported issues with stimulus audibility in a post-test questionnaire (“Did the sounds skip or not play?”) affecting more than one or two instances.

**1.3 Participant Exclusion Based on the Suprathreshold Trials in the Perceptual Discrimination Task**

We included the perceptual discrimination task as a control measure to account for possible group-level differences in sensitivity to prosodic stimuli. If groups differed in their ability to perceive subtle differences in prosodic contours, possible differences in the adaptation task could be explained by these low-level perceptual differences rather than by their adaptivity *per se*. Interspersed in the discrimination task were eight suprathreshold (i.e., very easy) stimuli that consisted of items that were six (vs. two) steps apart on the 11-step continuum. According to our predetermined cutoff of 75% accuracy on the suprathreshold discrimination trials, we removed 6 participants from the current autistic young adult group.

***Table S1****. Demographic information of participants who scored below 75% accuracy on suprathreshold trials in the perceptual discrimination task.*

| Group | Autistic adolescents | Autistic young adults | NA adolescents | NA young adults |
| --- | --- | --- | --- | --- |
| N | 7 | 6 | 3 | 3 |
| Mean age in years (SD) | 15.9 (1.37) | 25.98 (2.2) | 14.5 (1.13) | 20.7 (1.15) |
| Female/Male/Nonbinary | 3/4/0 | 2/4/0 | 2/1/0 | 1/1/1 |
| Mean SRS-2 Total T-score (SD) | 75.1 (6.09) | N/A | 42 (5.29) | N/A |
| Autism Quotient Total Scores | N/A | 33.66 (4.32) | N/A | N/A |

# Prosodic Continua

The stimuli were adapted from a study previously published by Xie et al. (2021). These consisted of six continuums in the form of “It's X-ing”, with six different verbs: 'booting', 'cooling', 'cooking', 'losing', 'muting', and 'moving'. These were based on the natural production of statements (e.g., “It's cooking”) and questions (e.g., “It's cooking?”) by a male native speaker of American English in his mid-20s. He was originally from Long Island, NY, and had spent over three years in the Upstate New York area at the time of recording.

The recordings (two tokens per item) were first segmented into three regions corresponding to three syllables (i.e., “It's | X- | ing”). To achieve the best resynthesis results, segmentation was based on a turning point in the F0 contour within the “X-ing” part, as well as segmental information, to delineate the last two syllables. The F0 of each region was sampled at 20 equally spaced time points and the measures from each time point were averaged across items to derive mean F0 contours for the statement and question. Similarly, the durations of each region were averaged across items by contour type. The mean F0 contours and durations were then derived by interpolating values within each region, and by manipulating the F0 and duration of each recording to match these interpolated values, using the pitch-synchronous overlap-and-add algorithm implemented in Praat (Boersma & Weenink, 2017). We used this technique to create steps 0 through 11 for each of the six verbs. (See Kurumada et al., (2018); Xie et al., (2021)) for details. All the training and test stimuli can be downloaded from <https://osf.io/p5v3k/>.

The six continua were normed with 60 native American English speakers. The results of this study can be found in Appendix III of Xie et al. (2021). Based on these results, we determined that steps 0 and 1 were indistinguishable. Step 0 was therefore omitted from the experimental stimuli, resulting in an 11-step continuum for each verb. Additionally, step 7 of the training tokens (booting, cooling, losing, muting and moving) sounded the most ambiguous between a question and a statement. In the current experiment, step 7 was presented during training and accompanied by “question” feedback.Click or tap here to enter text.Click or tap here to enter text.

# Screening based on the Discrimination Task: Data Analysis

# The analysis focused only on responses to the 76 standard trials, where an oddball stimulus was two steps away from the baseline (e.g., Step1-Step3-Step3). We predicted listeners’ trial-level response accuracy (1 = correct vs. 0 = incorrect) based on two fixed effect variables: autism condition (factorial, contrast-coded: autistic = 1, non-autistic = -1), age group (factorial, contrast-coded: young adults = 1, adolescents = -1), and an interaction term between them. We considered the maximal random effect structure justified by the design (Barr et al., 2013), i.e., participant and item (e.g., a pair of steps such as Step 1 and Step 3). The final model included random intercepts for participant and item, as well as by-item slopes for the autism * age group interaction.

Table S2 provides the model summary. There was no significant effect of any of the fixed effects, indicating that all groups demonstrated equivalent levels of accuracy. This finding supports the underlying logic of the adaptation experiment. In other words, any difference observed between groups in the adaptation results cannot be solely attributed to differences in perceptual accuracy.

***Table S2****. Fixed-effect estimates of the generalized linear mixed-effect model predicting the response accuracy in the discrimination task.*

|  | Estimate | Std. Error | z value | Pr (> \|z\|) |  |
| --- | --- | --- | --- | --- | --- |
| (Intercept) | 2.828 | .123 | 22.822 | < 2e-16 | ** |
| Autism (autistic = 1) | -.067 | .112 | -.604 | .546 |  |
| Age group (young adults = 1) | -.131 | .109 | -1.195 | .232 |  |
| Autism * Age group | .045 | .110 | .408 | .684 |  |

# Autism * Age Analysis with the 11-step Continuum

We constructed a mixed-effect logistic regression model to predict a trial-level response (Question = 1, Statement = 0). The model included 4 fixed effects and their interactions: autism condition (factorial; contrast coded: autistic = 1, non-autistic = -1), age group (factorial; contrast coded: young adults =1, adolescents = -1), block (factorial; contrast coded: post-test = 1, pre-test = −1), and continuum (numerical variable: 1–11; centered). Centering the continuum ensures that its interaction with the other two fixed effects is assessed at the center of the continuum, where the adaptation effect is expected to be most prominent. We used the maximal random effect structure justified by the data, i.e., by-participant random intercepts and slopes for block and continuum and the interaction between them. We then removed higher order interactions when a model failed to converge. The final model included by-participant random intercepts and slopes for block and continuum.

As in the main model reported in the manuscript, the main effects of the autism condition ($\hat{\beta}$ = .332, z = 3.241, *p* < .001) and its negative interaction with age group ($\hat{\beta}$ = -.234, z = -2.286, *p* < .022) were significant. These results suggest that autistic individuals are more likely than their non-autistic counterparts to provide "question" responses, particularly in the adolescent group. Across autism condition and age groups, more "question" responses were given for higher steps along the continuum ($\hat{\beta}$ = .1.576, z = 31.536, *p* < 2e-16) and in post-test than in pre-test ($\hat{\beta}$ = .495, z = 10.567, *p* < 2e-16), i.e., adaptation.

***Table S3****. Fixed-effect estimates of the generalized linear mixed-effect model predicting the categorization response (question = 1, statement = 0) in the adaptation task. Effects of importance are shaded in blue.*

|  | Estimate | Std. Error | z value | Pr (> \|z\|) |  |
| --- | --- | --- | --- | --- | --- |
| (Intercept) | .428 | .104 | 4.109 | 3.97e-05 | *** |
| Autism (autistic = 1) | .332 | .102 | 3.241 | .001 | ** |
| Age group (young adults = 1) | -.007 | .102 | -.076 | .939 |  |
| Block (post = 1) | .495 | .046 | 10.567 | < 2e-16 | *** |
| Continuum | 1.576 | .049 | 31.536 | < 2e-16 | *** |
| Autism * Age group | -.234 | .102 | -2.286 | .022 | * |
| Autism * Block | -.009 | .045 | -2.216 | .026 | * |
| Age group * Block | -.037 | .044 | -.833 | .404 |  |
| Autism * Continuum | -.044 | .044 | -.992 | .321 |  |
| Age group * Continuum | -.048 | .031 | -1.548 | .121 |  |
| Block * Continuum | -.209 | .029 | -7.128 |  |  |
| Autism * Age group * Block | .012 | .048 | .391 | 0.695 |  |
| Autism * Age group * Continuum | -.038 | .047 | -.812 | 0.416 |  |
| Autism * Block * Continuum | .057 | .017 | 3.365 | 0.0009 | *** |
| Age group * Block * Continuum | .03 | .017 | 1.725 | 0.084 | . |
| Autism * Age group* Block * continuum | .062 | .017 | 3.557 | 0.0004 | *** |

The effect of critical interest is the autism * block interaction, and the negative interaction means that the adaptive shift was smaller in autistic individuals than in non-autistic individuals ($\hat{\beta}$ = -.009, z = -2.216, *p* < .026). We note that the three-way interaction (autism * block * continuum) is also significant with a positive intercept ($\hat{\beta}$ = -.057, z = 3.365, *p* < .001). This suggests that autistic individuals showed a strong adaptation effect towards the higher end of the continuum. This can be seen in the pre- vs. post-test categorization functions in Figure 3 of the main manuscript. While pretest responses for steps 10 and 11 reached the ceiling in nonautistic groups, autistic groups showed some room for improvement. Additionally, the non-autistic groups increased their "question" responses toward the lower end of the continuum, most evidently in the non-autistic young adult group (the four-way interaction between autism, age group, block, and continuum, $\hat{\beta}$ = .062, z = 3.557, *p* < .001).

Finally, the model indicates a trend of age group * block * continuum interaction ($\hat{\beta}$ = .03, z = 1.725, *p* < .09). This indicates that young adults exhibited marginally larger adaptation effects in each autism condition group, especially for stimuli sampled from the higher end of the continuum. This corresponds to the marginal effect of the age group * block interaction reported in the main body of the manuscript and provides additional information that this effect was more pronounced at one end of the continuum than the other.

# Effects of Age and Autism Severity

To assess the predictive values of the numerical variables of AQ and age, we constructed two separate mixed-effect logistic regression models.

## Age

We constructed a mixed-effect logistic regression model to predict a trial-level response (Question = 1, Statement = 0) to stimuli from the mid-region of the continuum (Steps 5, 6, 7). The model included 3 fixed effects and their interactions: autism condition (factorial; contrast coded: autistic = 1, non-autistic = -1), age (in years, numeric), and block (factorial; contrast coded: post-test = 1, pre-test = −1). We used the maximal random effect structure justified by the data, i.e., by-participant random intercepts and slopes for block.

***Table S4****. Fixed-effect estimates of the generalized linear mixed-effect model predicting the categorization response (question = 1, statement = 0) in the adaptation task. This is the same model as the main by-group model reported in the main body of the manuscript, except that the factorial “age group” variable is replaced with the numeric variable of age. Effects of importance are shaded in blue.*

|  | Estimate | Std. Error | z value | Pr (> \|z\|) |  |
| --- | --- | --- | --- | --- | --- |
| (Intercept) | .316 | .087 | 3.622 | .0003 | *** |
| Autism (autistic = 1) | .229 | .087 | 2.637 | .008 | ** |
| Age (centered) | .002 | .019 | .121 | .903 |  |
| Block (post = 1) | .397 | .042 | 9.254 | < 2e-16 | *** |
| Autism * Age | -.032 | .019 | -1.661 | .096 | . |
| Autism * Block | -.094 | .042 | -2.221 | .026 | * |
| Age * Block | .01 | .009 | 1.117 | .263 |  |
| Autism * Age* Block | -.009 | .009 | -.962 | .336 |  |

The two-way negative interaction of interest—autism * block—is significant, as in the main model reported in the main body of the manuscript ($\hat{\beta}$ = -.094, z = -2.221, *p* < .03). However, there was no main effect of age ($\hat{\beta}$ = .002, z = .121) or its interaction with autism and block ($\hat{\beta}$ = -.009, z = -.962).

**5.2 AQ**

We constructed a mixed-effect logistic regression model to predict a trial-level response (Question = 1, Statement = 0) to stimuli from the mid-region of the continuum (Steps 5, 6, and 7). The model included 2 fixed effects and their interactions: block (factorial; contrast coded: post-test = 1, pre-test = −1), AQ total score (numeric, centered), and their interactions. We used the maximal random effect structure justified by the data, i.e., by-participant random intercepts and slopes for block.

The expected effect of block ($\hat{\beta}$ = .356, z = 5.097, *p* < .0001) was significant. However, neither the main effect of AQ ($\hat{\beta}$ = -.028, z = -1.432) nor its interaction with block ($\hat{\beta}$ = -.007, z = -.663) was significant. Figure S1 illustrates this, showing the predicted increase in responses to questions for each participant. Overall, AQ scores do not explain the variance in the degree of adaptation.

***Table S5****. Fixed-effect estimates of the generalized linear mixed-effect model predicting the categorization response (question = 1, statement = 0) in the adaptation task. This model uses data from autistic young adults only.*

|  | Estimate | Std. Error | z value | Pr (> \|z\|) |  |
| --- | --- | --- | --- | --- | --- |
| (Intercept) | .335 | .131 | 2.557 | 0.01 | * |
| Block (post = 1) | .356 | .069 | 5.097 | 3.45e-07 | *** |
| AQ (centered) | -.028 | .02 | -1.432 | 0.152 |  |
| Block * AQ | -.007 | .01 | -.663 | 0.507 |  |


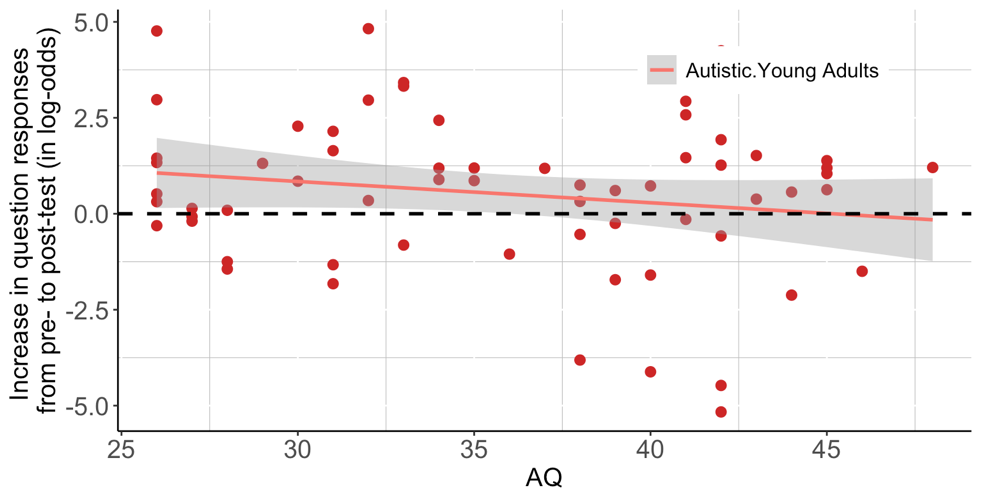


**Supplementary Figure 1*.*** *Increase in question responses from pre- to post-test (in log-odds), predicted in the generalized linear mixed-effects model on the effects of AQ and block. The x-axis represents the AQ total score, with a lower cutoff point of 26. Individual participants are represented by points, and the regression line was added using the “lm” function in the ggplot2 package. The smoother indicates the 95% confidence interval. The dashed line indicates a value of 0, showing that there was no difference before and after the training.*

**References**

Baron-Cohen, S., Wheelwright, S., Skinner, R., Martin, J., & Clubley, E. (2001). The autism-spectrum quotient (AQ): Evidence from Asperger syndrome/high-functioning autism, males and females, scientists and mathematicians. *Journal of Autism and Developmental Disorders*, *31*(1), 5–17. https://doi.org/10.1023/A:1005653411471

Barr, D. J., Levy, R., Scheepers, C., & Tily, H. J. (2013). Random effects structure for confirmatory hypothesis testing: Keep it maximal. *Journal of Memory and Language*, *68*(3), 255–278. https://doi.org/10.1016/j.jml.2012.11.001

Boersma, D. C., & Weenink, D. (2017). *Praat: Doing phonetics by computer (Version 6.0.29)* (6.0.29). http://www.praat.org/

Kurumada, C., Brown, M., & Tanenhaus, M. K. (2018). Effects of distributional information on categorization of prosodic contours. *Psychonomic Bulletin and Review*, *25*, 1153–1160.

Xie, X., Buxó-Lugo, A., & Kurumada, C. (2021). Encoding and decoding of meaning through structured variability in speech prosody. *Cognition*, *211*. https://doi.org/https://doi.org/10.1016/j.cognition.2021.104619
